# Ni-Cu Nanoparticles and Their Feasibility for Magnetic Hyperthermia

**DOI:** 10.3390/nano10101988

**Published:** 2020-10-09

**Authors:** Bianca P. Meneses-Brassea, Edgar A. Borrego, Dawn S. Blazer, Mohamed F. Sanad, Shirin Pourmiri, Denisse A. Gutierrez, Armando Varela-Ramirez, George C. Hadjipanayis, Ahmed A. El-Gendy

**Affiliations:** 1 Department of Physics, the University of Texas at El Paso (UTEP), El Paso, TX 79968, USA; bmenesesbr@miners.utep.edu (B.P.M.-B.); dsblazer@miners.utep.edu (D.S.B.); mfsanad@miners.utep.edu (M.F.S.); 2 Border Biomedical Research Center, Department of Biological Sciences, the University of Texas at El Paso, El Paso, TX 79968, USA; eaborregopu@miners.utep.edu (E.A.B.); dagutierrez5@utep.edu (D.A.G.); avarela2@utep.edu (A.V.-R.); 3 Department of Physics and Astronomy, University of Delaware, Newark, DE 19716, USA; pourmiri@udel.edu (S.P.); hadji@udel.edu (G.C.H.)

**Keywords:** Ni-Cu nanoparticles, hyperthermia, cytotoxicity

## Abstract

Ni-Cu nanoparticles have been synthesized by reducing Ni and Cu from metal precursors using a sol–gel route followed by annealing at 300 °C for 1, 2, 3, 6, 8, and 10 h for controlled self-regulating magnetic hyperthermia applications. Particle morphology and crystal structure revealed spherical nanoparticles with a cubic structure and an average size of 50, 60, 53, 87, and 87 nm for as-made and annealed samples at 300 °C for 1, 3, 6, and 10 h, respectively. Moreover, hysteresis loops indicated ferromagnetic behavior with saturation magnetization (Ms) ranging from 13–20 emu/g at 300 K. Additionally, Zero-filed cooled and field cooled (ZFC-FC) curves revealed that each sample contains superparamagnetic nanoparticles with a blocking temperature (T_B_) of 196–260 K. Their potential use for magnetic hyperthermia was tested under the therapeutic limits of an alternating magnetic field. The samples exhibited a heating rate ranging from 0.1 to 1.7 °C/min and a significant dissipated heating power measured as a specific absorption rate (SAR) of 6–80 W/g. The heating curves saturated after reaching the Curie temperature (Tc), ranging from 30–61 °C within the therapeutic temperature limit. An in vitro cytotoxicity test of these Ni-Cu samples in biological tissues was performed via exposing human breast cancer MDA-MB231 cells to a gradient of concentrations of the sample with 53 nm particles (annealed at 300 °C for 3 h) and reviewing their cytotoxic effects. For low concentrations, this sample showed no toxic effects to the cells, revealing its biocompatibility to be used in the future for in vitro/in vivo magnetic hyperthermia treatment of cancer.

## 1. Introduction

Magnetic nanoparticle hyperthermia (MNH) is a method that has been developed to cure localized cancer as an addition to the existing methods to cure cancer, such as chemotherapy and radiotherapy [1,2,3,4]. MNH can treat the disease by injecting magnetic nanoparticles at the tumor site and allowing them to heat the localized tumors with the use of an external alternating magnetic field (AMF). The AMF causes the nanoparticles to absorb energy from the applied field and causes them to heat up, destroying the cancer cells within the therapeutic temperature range of 42–46 °C [4,5,6,7,8,9]. If the temperature goes beyond this range, heat-induced shock might occur, which could also damage healthy cells. Therefore, in the case of MNH using iron oxide nanoparticles with a Curie temperature (Tc) much higher than 42 °C, the regulation of energy absorption is achieved, for instance, by decreasing the field amplitude when 42 °C is reached. In order to overcome this issue, the Curie temperature (T_C_) of the injected nanoparticles maybe in this temperature range in order for the energy absorption to decrease and thus reduce the effect of overheating [9,10]. Then the heat will automatically stop due to the ferromagnetic to paramagnetic transition. This is called self-regulating magnetic hyperthermia. The purpose of this study was to synthesize biocompatible magnetic materials that have unique magnetic properties within the safety limit of the magnetic field, its frequency, and temperature. Herein, we synthesized Ni-Cu nanoparticles with a T_C_ of 27–77 °C (300–350 K). Ni-Cu bimetallic nanoparticles have attracted attention due to their superior electronic and magnetic properties at various temperatures [10,11,12,13,14,15]. Depending on the concentration of copper, the Ni-Cu system may demonstrate ferromagnetic or paramagnetic properties. In addition, due to their enhanced resistance to corrosion and their superior chemical and physical stability, researchers commonly use Ni-Cu alloys as thermocouple strips, resistances in electrical applications, heat resistant wires, etc. [11]. A recent study demonstrated the use of carbon nanotubes incorporated with Ni-Cu nanoparticles for the generation of hydrogen gas [12]. Moreover, since Ni-Cu bimetallic nanoparticles are chemically stable, biocompatible, and demonstrate the desired magnetic properties, they are perfect candidates for targeted drug delivery for cancer diagnosis and therapy [12,13]. An important application of Ni-Cu magnetic nanoparticles is in cancer therapeutics as materials for hyperthermia treatment [6]. Magnetic nanoclusters, which contain small groups of individual nanoparticles, can show ferromagnetic behavior with a T_C_ in the therapeutic temperature range and enhanced dispersion in solution under an applied AC field. This will lead to homogeneous energy absorption by the particles in solution, revealing high heating power known as the specific absorption rate (SAR) under the safety limit of an AC field of 5 × 10^9^ A/(ms), which is defined by *f xH*_0_, where *f* is the frequency and *H*_0_ the field amplitude [16,17,18,19,20]. Ni-Cu alloys are usually synthesized in the bulk form. However, a few strategies, such as mechanical synthesis, hydrothermal, electrodeposition, pulsed deposition, and microwave methods, have been employed to synthesize these systems in the nano-form [5,6,10,11,12]. Among them, the sol–gel method is a groundbreaking and straightforward technique to create such compounds, but most of this published work did not succeed in overcoming the barrier in in vivo studies due to the agglomeration of those particles in solutions and their weak magnetic properties that led to insufficient heating power to destroy the cancer cells [13,14].

In this study, we synthesized Ni-Cu nanoparticles by a sol–gel wet chemical process followed by annealing. The Ni-Cu nanoparticles presented demonstrated an improved dispersion in solution, providing an enhanced heating power under an applied AC magnetic field compared to previous work in Ni-Cu systems [11,12,13,14,15]. The morphology and composition of the nanoparticles were observed using a transmission electron microscope (TEM), scanning electron microscope (SEM), and X-ray diffraction (XRD). Lastly, the magnetic properties and the in vitro cytotoxicity of the prepared nanoparticles were assessed to evaluate their potential use in magnetic hyperthermia therapy.

## 2. Materials and Methods

All the materials used to synthesize the Ni-Cu nanoparticles were obtained from Thermo Fisher Scientific (Rochester, NY, USA). Following the sol–gel method by Stergar et al. [12], our samples were synthesized by mixing and stirring 0.2 g of copper (I) chloride and 0.8 g of nickel (II) acetate tetra-hydrate in 150 mL of ethanol at 70 °C. Parallel to this mixture, 1.0 g of sodium hydroxide dissolved in 50 mL of distilled water and 2.5 g of sodium citrate dissolved in 75 mL of distilled water were added slowly to the mixture for 1–2 min separately. After that, a solution of 2 mL of hydrazine mixed with 0.5 mL of distilled water was added to the mixture without further heating. Then 1.5 g of sodium borohydride dissolved in 15 mL of distilled water were added into the mixture while stirring for 30 min. The samples were collected by using filter paper and afterwards several ethanol wash steps were performed. Then part of the as-made sample was annealed at 300 °C for 1, 2, 3, 6, or 10 h. Subsequently, the crystal structure of the samples was measured by an X-ray diffractometer (P-Analytical X’PERT MPD instrument) (Malvern Panalytical Ltd., Malvern, UK) and the particle morphology by scanning electron microscopy (FESEM JEOL6340 electron microscope) and transmission electron microscopy (HTEM) (JEOL-JEM-1230). A vibrating sample magnetometer (VSM) (Quantum Design, 3T Versalab) was used to study the magnetic properties. The feasibility of hyperthermia of 5 mg Ni-Cu nanoparticles dispersed in 1 mL of water was measured by using a G2-D5 Series Multi-mode 1500W Driver from Nanoscale Biomagnetics. Each sample solution was placed into a G2-D5 coil, where the alternating magnetic field and frequencies were applied. The induced temperature of the solution was measured and recorded using a fiber optic sensor. Cytotoxicity assays were performed in human triple-negative breast cancer MDA-MB231 cells acquired from the American Tissue Culture Collection (ATCC). The MDA-MB231 cells were cultured by using DMEM culture media (from Corning) supplemented with 10% heat-inactivated fetal bovine serum (Hyclone) and 1× antibiotics; 100 U/mL penicillin, and 100 μg/mL streptomycin (Life Technologies). Before preparing any experiment, the cell viability was measured by using the propodeum iodide exclusion assay and flow cytometry, as previously detailed. Only cultures with a viability of 95% or higher were used [21]. Consistently, the cells were incubated at 37 °C under a humidified 5% carbon dioxide (CO_2_) atmosphere using a typical water-jacketed incubator. The cytotoxicity analysis was performed by using the differential nuclear staining (DNS) assay [22,23]. Cells growing exponentially, around 60–70% confluence, were collected and seeded on a 96-well plate format at a density of between 2500 and 10,000 cells/well in 100 µl of culture media, followed by overnight incubation. Subsequently, the cells were exposed for 1, 2, 3, and 6 days to a concentration gradient of magnetic nanoparticles. At each indicated incubation time, cells were stained with 1 µg/mL of Hoechst and propidium iodide (PI) reagents, incubated for 2 h, and analyzed via an IN Cell 2000 bio imager system. In addition, untreated cells and solvent-treated (PBS) cells, as well as 1 mM H_2_O_2_-treated cells incubated for 24 h, were included as a positive control of cytotoxicity. Four contiguous images were acquired per well, forming 2 × 2 montages with a 10× objective with two individual fluorescent channels (Hoechst and PI emission signals). Image acquisition and segmentation were achieved by using the IN Cell Analyzer 2000 bioimager system and the IN Cell Analyzer Workstation 3.2 software (GE Healthcare, Pittsburg, PA, USA), respectively, to obtain both the living and dead cell subpopulations per individual well [24,25]. The results are depicted as an average, with their corresponding standard deviation, to denote the experimental (biological and technical) variability. A two-tailed paired Student’s *t*-test was utilized to define the statistical significance of the two experimental samples.

## 3. Results and Discussion

The Ni-Cu nanoparticles were synthesized by using a wet chemistry method with a 4:1 Ni:Cu precursor ratio, as discussed before. The sample was then annealed at 300 °C for 1, 2, 3, 6, or 10 h, in order to obtain the required magnetic order. The XRD pattern (Figure S1) of the as-made sample shows a mixture of face centered cubic (FCC) structures for Ni_0.2_Cu_3.8_ (Cu-rich phase, Ref. JCPDS 98-062-8549) as the major phase and an FCC structure of Ni_3.68_Cu_0.32_ (Ni-rich phase, Ref. JCPDS 98-062-8545) as a minor phase. By annealing at 300 °C for 1, 2, 3, 6, or 10 h, the ratio of Ni_3.68_Cu_0.32_ (Ni-rich phase) to Ni_0.2_Cu_3.8_ (Cu-rich phase) changed. With annealing, the Cu-rich phase increased at the expense of the Ni-rich phase. Using the Gaussian peak analysis, we estimated the ratio of Ni-rich to Cu-rich phases to be 59 to 41%, 54 to 46%, 52 to 48%, 51.8 to 48.2%, 52.6 to 47.4%, and 49 to 51% for the as-made and annealed samples at 300 °C for 1, 2, 3, 6, and 10 h, respectively. The width of the characteristic diffraction peaks was affected by the average crystalline size (D) of the particles, which was calculated using the Scherrer equation [6] (D = 0.93λ/Δ2θ cosθ, where λ is the wavelength of the applied radiation (Cu Ka), Δ2θ is the full width at half maximum of the diffraction peak, and θ is the Bragg angle), for all the samples, as shown in Figure S1. The average crystalline size of the Ni-rich phase increased from 3 nm to 9–10 nm with increasing annealing time, while the average size of the Cu-rich phase was nearly constant at 28–30 nm.

**Figure 1:**
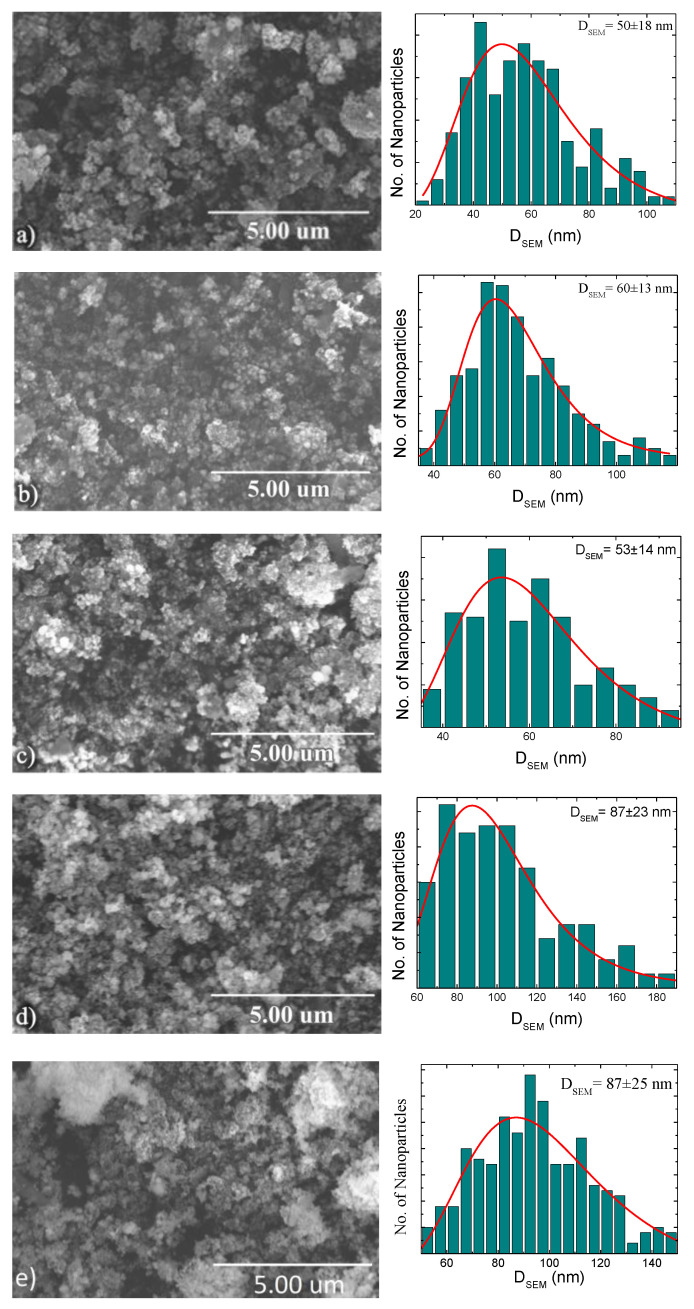
SEM and size distribution of Ni*_x_*Cu_4−*x*_ nanoparticles (measured from SEM images) annealed at 300 °C for: (**a**) 0 h, (**b**) 1 h, (**c**) 3 h, (**d**) 6 h, and (**e**) 10 h. Scale bar = 5 µm.

**Figure 2:**
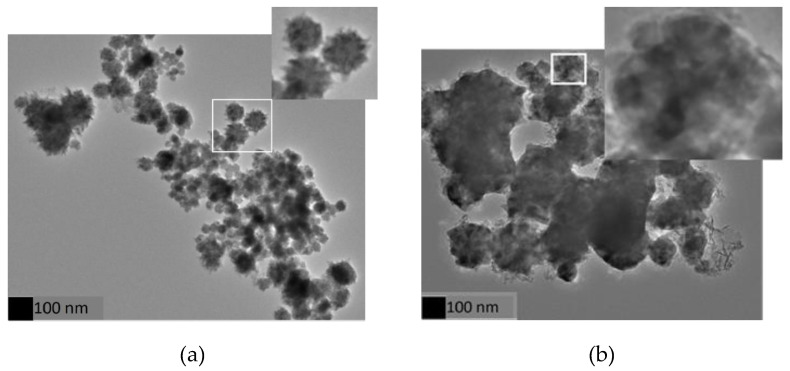
TEM image of the Ni*_x_*Cu_4−*x*_ nanoparticles revealing the formation of nanoclusters for (**a**) the as-made and (**b**) the annealed samples.

Both scanning and transmission electron microscopy (SEM and TEM) were used to study the morphology of the formed nanoparticles. Figure 1 shows SEM images of the particle sizes and their distribution. We believe that these SEM sphere-like particles are particle clusters consisting of smaller nanoparticles (the resolution of our SEM images is in the range of 20–30 nm). The cluster size distribution calculated from the SEM images yields sizes of 50 ± 18, 60 ± 13, 53 ± 14, 87 ± 23, and 87 ± 25 nm for as-made and annealed samples at 300 °C for 1, 3, 6, and 10 h, respectively. It is clear from Figure 1 that the as-made, annealed samples for 1 and 3 h show a narrower size distribution with a standard deviation of less than 50 % among all the samples. Therefore, a narrower size distribution leads to the disaggregation of the nanoparticles in solution, producing a homogeneous heat distribution when they are injected to the cells. On the other hand, for the 6 and 10 h annealed samples, a more flat and spread out distribution suggests that the particles deviated significantly from their mean value. As can be seen in Figure 2, TEM images confirm the formation of clusters, showing bigger particles containing smaller nanoparticles, both in the as-made and annealed samples.

**Figure 3:**
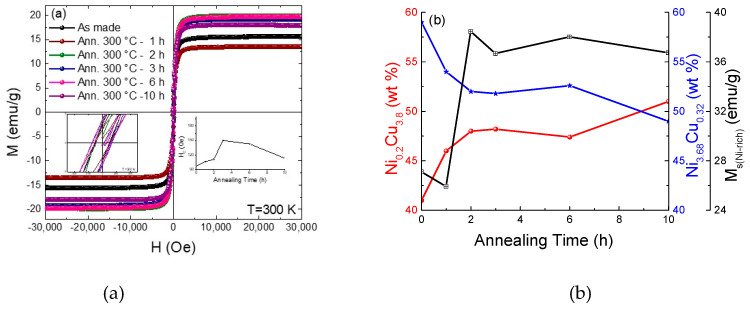
(**a**) Hysteresis loops of the synthesized Ni-Cu nanoparticles at T = 300 K. Inset figures represent the dependence of *H_c_* and *M_r_* on annealing time. (**b**) Ni-rich/Cu-rich phase ratios and Ni-rich normalized saturation magnetization dependence on annealing time.

To understand the magnetic properties of the synthesized Ni-Cu nanoparticles, hysteresis loops at 300 K with an external magnetic field up to 3T were measured, as shown in Figure 3a. It is clear from Figure 3a (insets) that the presence of remnant magnetization (*M_r_*) and coercivity (*H_c_*) for the as-made and annealed samples revealed ferromagnetic-like behavior at room temperature for a significant fraction of the Ni-Cu nanoparticles, which is attributed mostly to the Ni-rich phase. Moreover, the samples show saturation magnetization (M_S_) of 16, 14, 20, 19, 20, and 18 emu/g for the as-made and annealed samples of 1, 2, 3, 6, and 10 h, respectively. In Figure 3a (right inset), *H_c_* shows an increase with annealing time up to 2 h, then slightly decreases for longer annealing times, following the size dependence of Ni-rich particles on annealing time. As seen from Figure 3b, the samples show an observable increase in *M_s_* when increasing the annealing time from 1 to 2 h, then it almost saturates at longer annealing times. By using the XRD data on the estimated ratio of the Ni-rich/Cu-rich (magnetic/non-magnetic) phases’ dependence on annealing time, as shown in Figure 3b, one can estimate the normalized saturation magnetization based on the amount of Ni-rich magnetic phase. Figure 3b confirms that the samples show observable increases in normalized *M_s_* for the Ni-rich phase with increasing annealing time from 1 to 2 h, then it almost saturates at longer annealing times. Based on the XRD and SEM data, each particle contains a group of small nanoparticles (clusters), which may show a superparamagnetic-like behavior. To further understand this behavior, the *M vs. T* data in zero-field-cooled (ZFC) and field-cooled (FC) samples were measured at 200 Oe from 50 to 400 K (Figure S2). Figure S2 shows broad peaks in the ZFC data, suggesting a distribution of blocking temperature (*T_B_*) corresponding to the maxima of ZFC curves in the range between 196 K and 260 K; *T_B_* depends on the particle volume and anisotropy constant (*KV = 25k_B_T_B_*, where *K* is the magnetic anisotropy, V is the volume of the particles, and k_B_ is the Boltzmann constant [16,17]). The data show clearly that the average blocking temperature increases with the annealing time, consistent with the observations that the grain size of the Ni-rich phase increases with annealing time. These observations can explain the increase in *M_s_* with annealing time. The as-made Ni-rich clusters are very small and most of them are superparamagnetic and this explains the smaller value of *M_s_*; with annealing, the Ni-rich particles get bigger and their magnetization is larger. Moreover, since we have a mixture of superparamagnetic and ferromagnetic particles, it was difficult to determine the exact value of T_C_. Therefore, we estimated it using the Curie–Weiss law [26], revealing values in the range of 300–350 K for all the samples.

In contrast to all the previous measurements of powder samples, to test the feasibility of Ni-Cu nanoparticles for the hyperthermia treatment of cancer, each nanoparticle sample was dispersed in a water solution with a 5 mg/mL concentration; due to the small size of the nanoparticles, they were highly dispersed in their water solutions. Then, each nanoparticle solution was subjected to an AC magnetic field at different amplitudes *H* and frequency *f*, from 100 to 500 Oe and 144, 164, and 304 kHz, respectively. Using a fiber optic sensor, measurements of temperature versus the exposure time (*T-t*) were recorded, as shown in Figure S3a–f, at different fields *H* of 100–500 Oe and at a frequency *f* of 144 kHz. Measurements were done at 164 and 304 kHz and can be found in the Supplementary Materials of this paper (Figures S4 and S5). It can be observed that the figures only portray the magnetic field intensities at which heating occurred and the last one, where there was no heating, as a reference. It can be observed that increasing the amplitude *H* and *f* of the applied AC field leads to higher temperatures due to the faster rotation of the magnetic moments and the nanoparticles themselves. To be specific, the data can be analyzed in the format of rising temperature with time (dT/dt) under an applied AC magnetic field at different *H* and *f* (Figure S6a–c). It is clear from Figure S6 that dT/dt increases when *H* and *f* increase. The annealed sample for 2 h shows the highest increase in dT/dt to be 1.7 °C/min at 400 Oe and 304 kHz. Therefore, we can determine the exposure time and control the hyperthermia process by knowing dT/dt of the used nanoparticles. For example, in order to use the annealed sample of 2 h for hyperthermia, it will take 25 min to reach 42 °C and then start to kill the cancer cells. In addition, we have observed that the T-t curves (Figures S3–S5) start to saturate after reaching a critical temperature, which will stop the heating automatically due to the ferromagnetic–paramagnetic transition. This property of the nanoparticles makes them self-regulated heat mediators for hyperthermia. 

**Figure 4:**
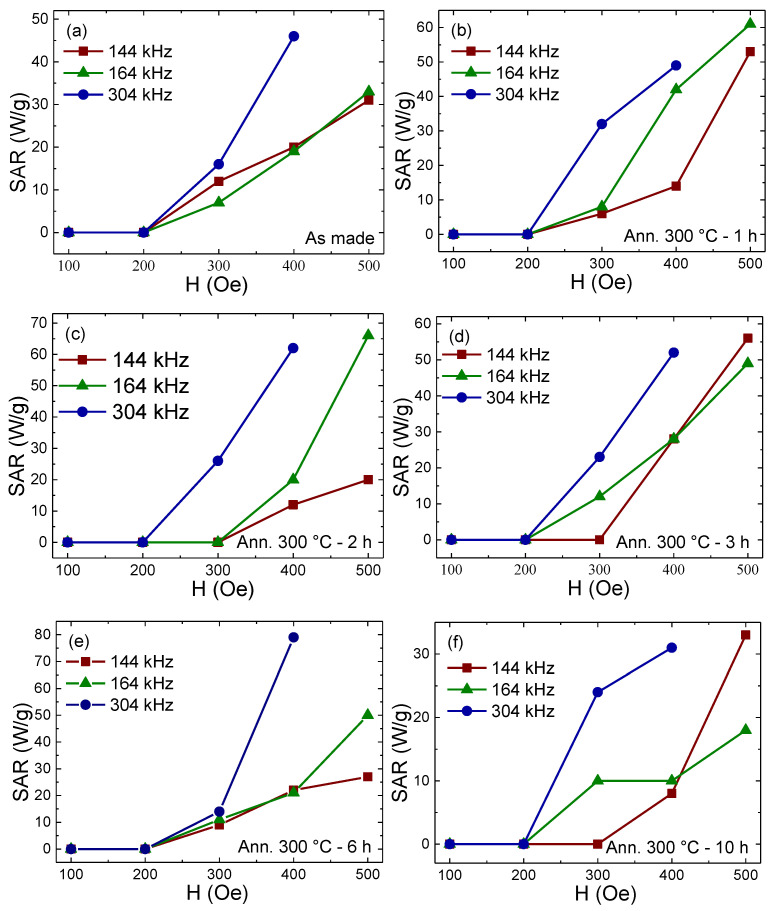
Specific absorption rate (SAR) dependence on applied AC magnetic fields H at different frequencies f for samples (**a**) as-made or annealed at 300 °C for (**b**) 1, (**c**) 2, (**d**) 3, (**e**) 6, and (**f**) 10 h (the points are connected as guidance for the eye).

The induced heating power per unit mass of the sample, known as specific absorption rate (SAR), was calculated from the initial slope of T-t curves (Figures S3–S5) [6,7,8]. Figure 4 shows the dependence of the samples’ SAR on the applied AC magnetic fields *H* at different frequencies *f*. It is clear that the annealed sample of 6 h yields the highest SAR, with 80 W/g when submitted to an AC magnetic field of 400 Oe (about 30 kA/m) and 304 kHz, consistent with the larger value of Hc. The other SAR values at lower fields and frequencies also generate enough heat, which matches the aim of this work to produce enough heat to kill the cancer cells under the safety limit of the AMF. These values are higher than those for all the published research of Ni-Cu nanoparticles [14]. In addition, the SAR values of the Ni-Cu system are lower than those of Fe or Fe_3_O_4_ due to the lower magnetization of Ni-Cu compared to those of strongly magnetic nanoparticles; however, the Ni-Cu system has self-regulating properties that do not exist in those strongly magnetic particles. Nevertheless, in future work, the SAR values can be further enhanced by surface functionalization of the nanoparticles that will lead to higher nanoparticle dispersion [14].

**Figure 5:**
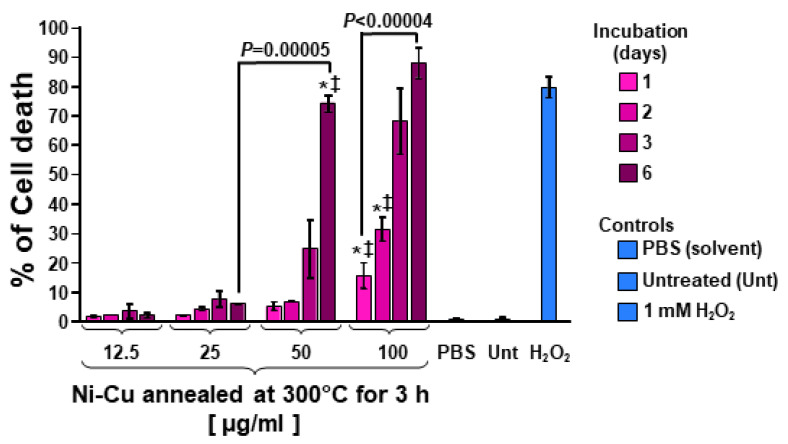
Ni-Cu magnetic nanoparticles inflict cytotoxicity on human breast MDA-MB231 cells in a dose-dependent manner. These nanoparticles displayed a size of 53 nm and were annealed at 300 °C for 3 h. Cells were incubated for 1, 2, 3, and 6 days with a nanoparticle concentration gradient (in μg/mL). Cellular cytotoxicity (*y*-axis) was quantified by using the differential nuclear staining assay (DNS: Hoechst and propidium iodide (PI)) and a bioimager system. PBS-treated (1% v/v) cells were used as a solvent control. Untreated (Unt) cells were included as a negative control. In contrast, cells exposed for 24 h to 1 mM of H_2_O_2_ were incorporated as a positive control for cytotoxicity. Data acquisition and analyses were accomplished via IN Cell Analyzer Workstation 3.2 software (GE Healthcare). When comparing an experimental sample with PBS-treated (*) or untreated cells (‡), the *p*-values were both <0.05.

To explore the cytotoxic properties of these Ni-Cu nanoparticles in biological tissues, we exposed human breast cancer MDA-MB231 cells to a concentration gradient of the nanoparticles with a size of 53 nm, which were annealed at 300 °C for 3 h, to review their cytotoxic effects (Figure 5).

The potential cytotoxic activity of Ni-Cu nanoparticles (sample with a size of 53 nm and annealed at 300 °C for 3 h) against human triple-negative breast cancer MDA-MB231 cells was performed by using the live-cell differential nuclear staining (DNS) assay via a high-throughput screening strategy. As shown in Figure 5, the Ni-Cu nanoparticles (sample with size of 53 nm and annealed at 300 °C for 3 h) exhibited non-toxic activity on cancer cells for low concentrations of 12.5 and 25 μg/mL for 1 to 6 days of incubation. Moreover, the cytotoxic values were similar to those observed for untreated and solvent control cells (<5%); almost no cytotoxicity was detected. In higher concentrations, such as 50 and 100 μg/mL, a cytotoxic effect was seen (Figure 5). When 50 µg/mL of the Ni-Cu sample were added to the cells, after 3 days of incubation, 24.6% of the cells were dead, whereas when 100 µg/mL were added to the cells, the cytotoxic effect was detected earlier at 1 day of incubation, with 15.4% dead cells. This toxic effect was significantly more accentuated when the nanoparticles were incubated for 6 days, with 88.1% dead cells (*p* < 0.00004). These results demonstrate that the newly synthesized Ni-Cu nanoparticles are inoffensive to the cell environment at low concentrations (12.5 to 25 μg/mL), which make them attractive for their potential use in magnetic hyperthermia.

## 4. Conclusions

To conclude, we have successfully synthesized Ni-Cu nanoparticles via a simple wet chemical method. The samples show spherical morphology with a cubic structure and an average particle size in the range of 50 to 80 nm. The as-made particles show a mixture of Ni-rich and Cu-rich phases, with the content of the Cu-rich phase increasing with annealing. The XRD, TEM, and M vs. T data suggest that the larger particles are clusters consisting of smaller nanoparticles with a size in the range of 3–9 nm. The broad ZFC/FC curves suggest superparamagnetic-like behavior with different blocking temperatures and average blocking temperatures that vary with the grain size. The magnetic properties of the prepared nanoparticles reveal ferromagnetic-like behavior at room temperature, indicating that a significant number of Ni-rich Ni-Cu particles (with a larger size) have a blocking temperature above room temperature with coercivity (Hc) of 97, 106, 111, 150, 143, and 113 Oe for the as-made and annealed samples of 1, 2, 3, 6, and 10 h, respectively. To examine their applicability for magnetic hyperthermia, the Ni-Cu nanoparticle powders were dispersed in water solutions. The nanoparticles showed feasibility for hyperthermia at different AMF strengths and frequencies. The heating power of our Ni-Cu nanoparticles exhibits higher values than most of the values found in the literature [14]. The cytotoxicity exerted by lower concentrations of Ni-Cu nanoparticles on human breast cancer cells after 6 days of exposure was negligible. Thus, the obtained results open new routes of using Ni-Cu nanoparticles for in vitro/in vivo studies of magnetic hyperthermia. 

## Supplementary Materials

The following are available online at https://www.mdpi.com/2079-4991/10/10/1988/s1, XRD pattern and crystallite size of Ni-Cu nanoclusters for as made and annealed at 300 °C for 1 h, 2 h, 3 h, 6 h, and 10 h. Figure S2: Magnetization dependence on temperature (MxT) at H = 200 Oe for as made and annealed Ni-Cu nanoclusters at 300 °C for 1, 2, 6, 10 h, Figure S3: Temperature vs Time at different AC magnetic fields at 144 kHz for samples (a) as made, annealed at 300 °C for (b) 1, (c) 2, (d) 3, (e) 6, and (f) 10 h, Figure S4: Temperature vs Time at different AC magnetic fields at 164 kHz for samples (a) as made, annealed at 300 °C for (b) 1, (c) 2, (d) 3, (e) 6, and (f) 10 h, Figure S5: Temperature vs Time at different AC magnetic fields at 304 kHz for samples (a) as made, annealed at 300 °C for (b) 1, (c) 2, (d) 3, (e) 6, and (f) 10 h, Figure S6: Change in heating rate dependence on applied AC magnetic fields H of Ni-Cu nanoclusters for as made and annealed samples at 300 °C at different frequencies f for (a) 144 kHz , (b) 164 kHz, and (c) 304 kHz.
