# Supplementary material for: Ni-Cu Nanoparticles and Their Feasibility for Magnetic Hyperthermia
Source: Nanomaterials (Basel). 2020 Oct 9;10(10):1988. doi: 10.3390/nano10101988 (PMC7599664; doi:10.3390/nano10101988)
Supplement: Supplementary file 1 [file nanomaterials-10-01988-s001.pdf]

Ni-Cu Nanoparticles and Their Feasibility for Magnetic Hyperthermia

Bianca P. Meneses-Brassea [1], Edgar A. Borrego [2], Dawn S. Blazer [1], Mohamed F. Sanad [1], Shirin Pourmiri [3], Denisse A. Gutierrez [2], Armando Varela-Ramirez [2], George C. Hadjipanayis [3] and Ahmed A. El-Gendy [1,*]

[1] Department of Physics, the University of Texas at El Paso (UTEP), El Paso, TX 79968, USA; bmenesesbr@miners.utep.edu (B.P.M.-B.); dsblazer@miners.utep.edu (D.S.B.); mfsanad@miners.utep.edu (M.F.S.)

[2] Department of Biological Sciences, Border Biomedical Research Center, the University of Texas at El Paso, El Paso, TX 79968, USA; eaborregopu@miners.utep.edu (E.A.B.); dagutierrez5@utep.edu (D.A.G.); avarela2@utep.edu (A.V.-R.)

[3] Department of Physics and Astronomy, University of Delaware, Newark, DE 19716, USA; pourmiri@udel.edu (S.P.); hadji@udel.edu (G.C.H.)

* Correspondence: aelgendy@utep.edu

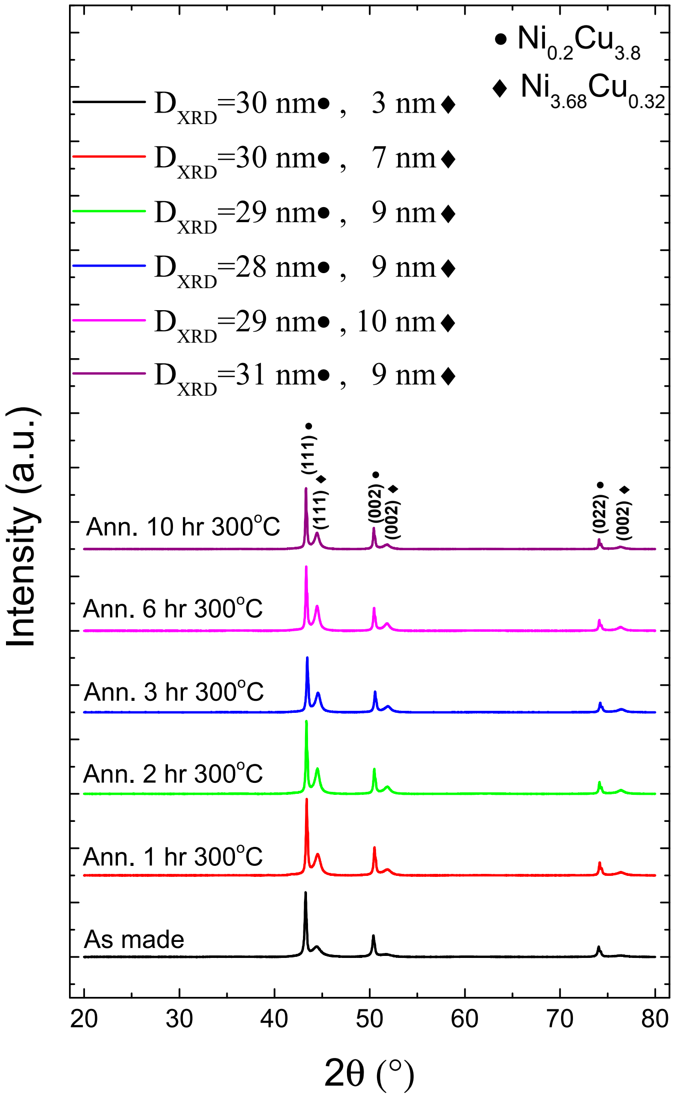

Figure S1. XRD pattern and crystallite size of Ni-Cu nanoclusters for as made and annealed at 300 °C for 1 hr, 2 hr, 3 hr, 6 hr, and 10 hr.

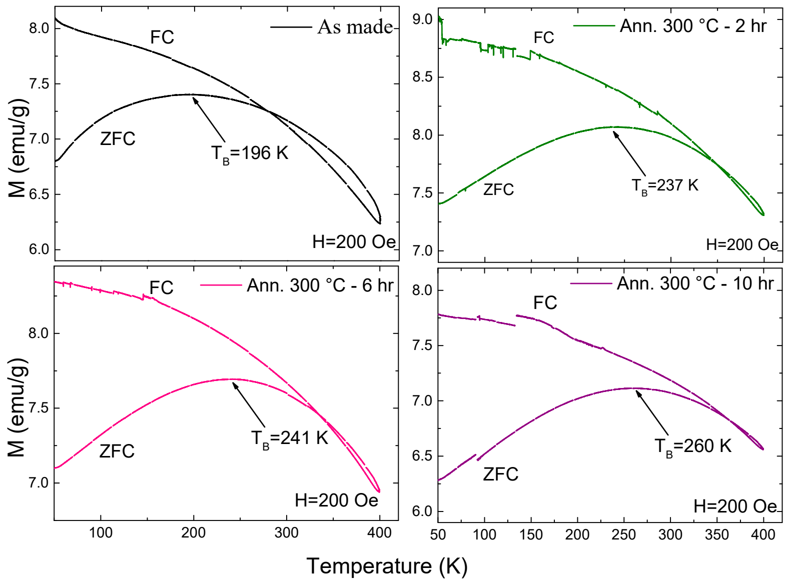

Figure S2. Magnetization dependence on temperature (MxT) at H=200 Oe for as made and annealed Ni-Cu nanoclusters at 300°C for 1, 2, 6, 10 hr.

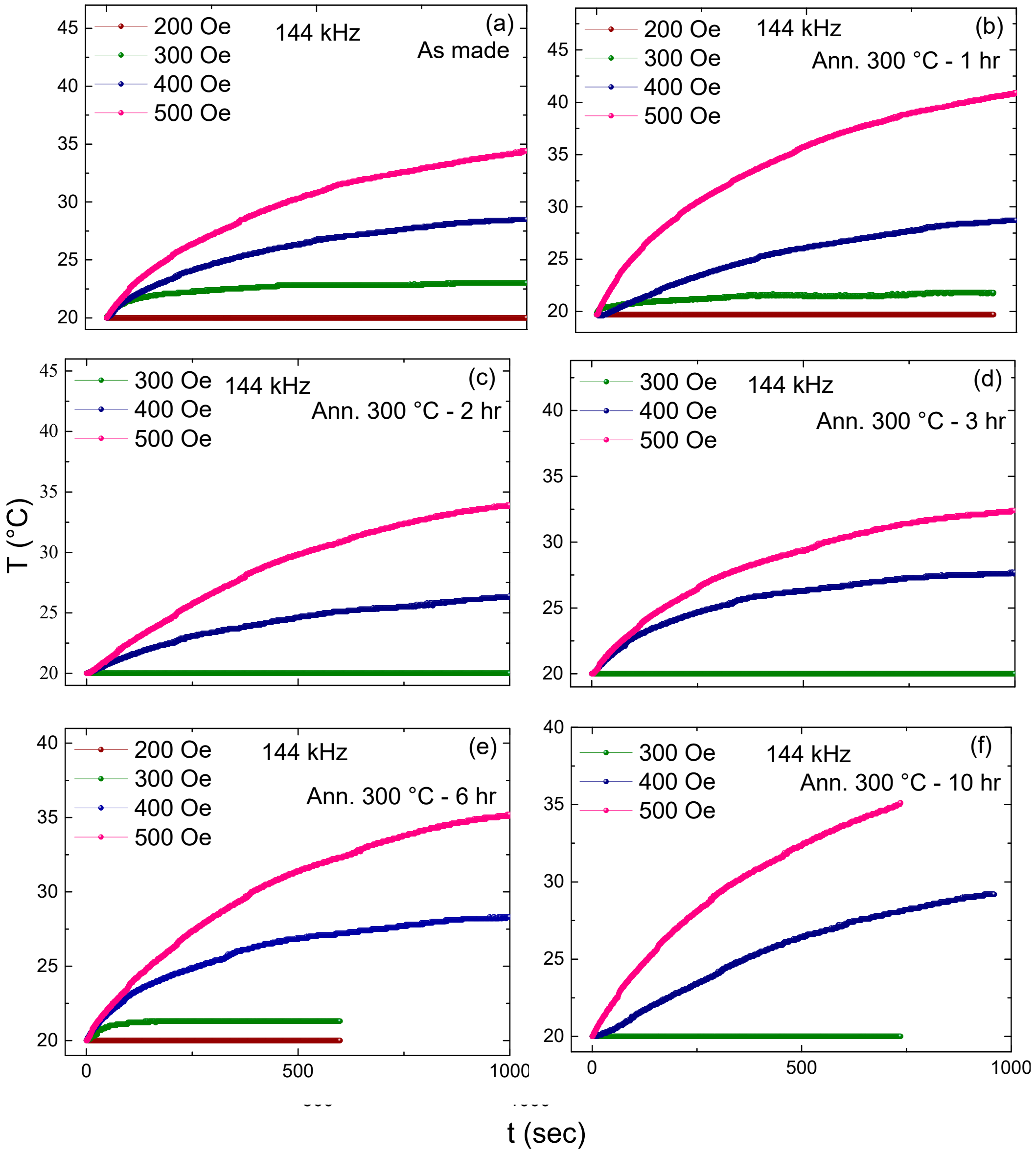

Figure S3. Temperature vs Time at different AC magnetic fields at 144 kHz for samples (a) as made, annealed at 300 °C for b) 1, c) 2, d) 3, e) 6, and f) 10 hr.

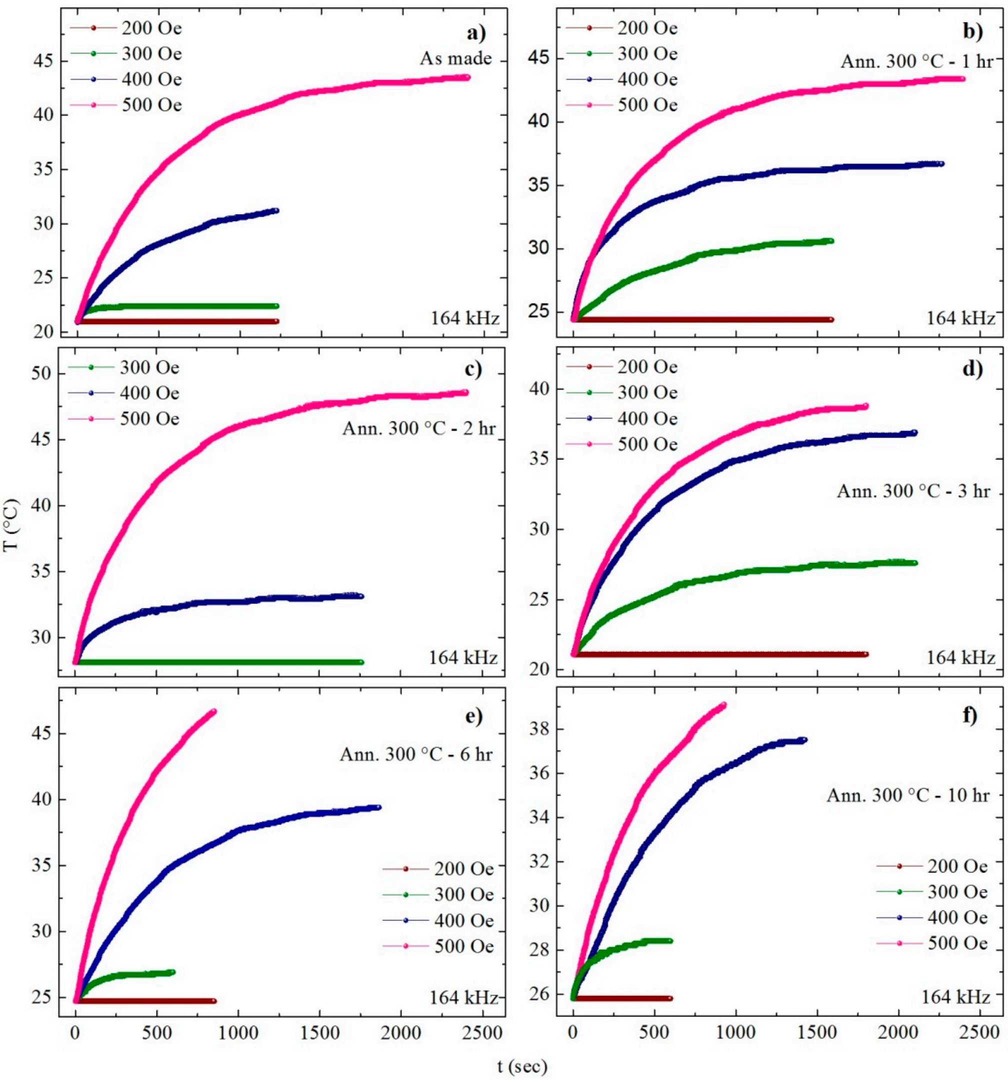

Figure S4. Temperature vs Time at different AC magnetic fields at 164 kHz for samples (a) as made, annealed at 300 °C for b) 1, c) 2, d) 3, e) 6, and f) 10 hr.

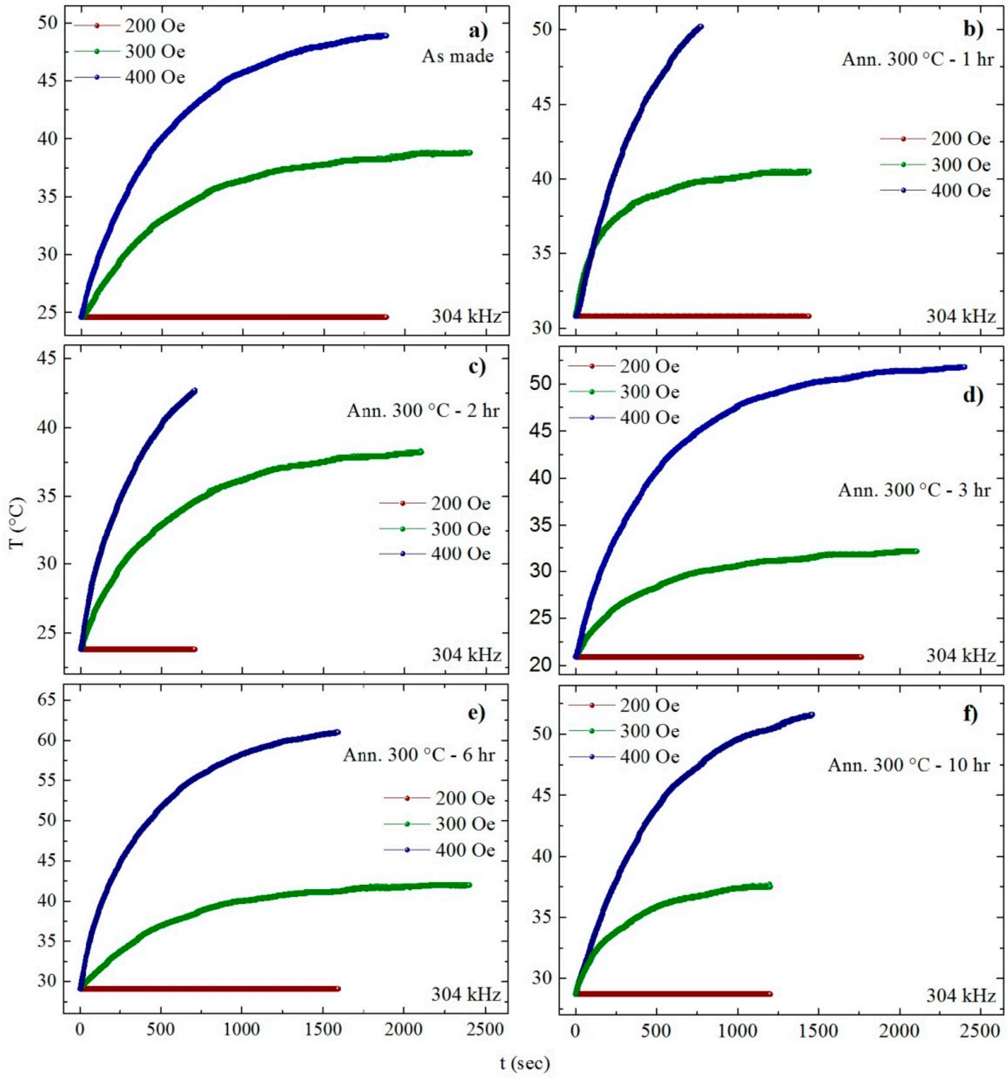

Figure S5. Temperature vs Time at different AC magnetic fields at 304 kHz for samples (a) as made, annealed at 300 °C for b) 1, c) 2, d) 3, e) 6, and f) 10 hr.

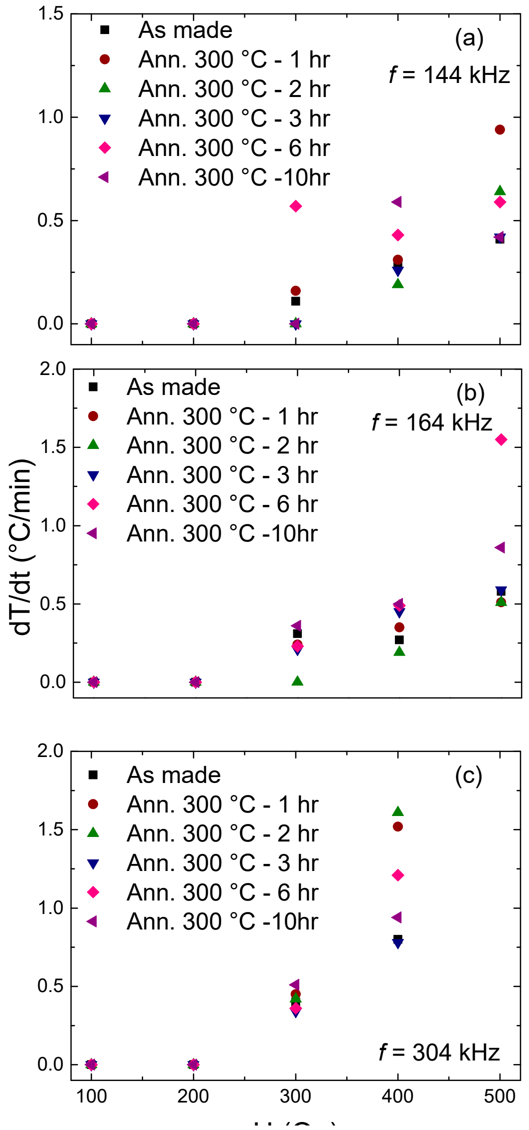

Figure S6. Change in heating rate dependence on applied AC magnetic fields *H* of Ni-Cu nanoclusters for as made and annealed samples at 300 °C.at different frequencies *f* for (a) 144 kHz , b) 164 kHz, and c) 304 kHz.
